# Clinical Outcomes of Piperacillin/Tazobactam Treatment in Outpatient Parenteral Antimicrobial Therapy (OPAT) Programs: Comparison of Two Models of Opat Care

**DOI:** 10.3390/pharmaceutics17111429

**Published:** 2025-11-04

**Authors:** Santiago J. Lora-Escobar, Laura Herrera-Hidalgo, Nerea Castillo-Fernández, Zaira R. Palacios-Baena, Rafael Luque-Márquez, Arístides De Alarcón, Ana Belén Guisado-Gil, Belén Gutierrez-Gutierrez, María Dolores Navarro-Amuedo, Julia Praena-Segovia, Marta Mejías Trueba, Juan Manuel Carmona-Caballero, José Manuel Sánchez Oliva, María Victoria Gil-Navarro, Luis E. López-Cortés

**Affiliations:** 1 Unidad de Gestión Clínica de Farmacia, Instituto de Biomedicina de Sevilla (IBiS), Hospital Universitario Virgen del Rocío, 41013 Seville, Spain; 2 Unidad Clínica de Enfermedades Infecciosas, Microbiología y Parasitología (UCEIMP), Instituto de Biomedicina de Sevilla (IBiS), Hospital Universitario Virgen del Rocío, 41013 Seville, Spain; 3 Centro de Investigación en Red de Enfermedades Infecciosas (CIBERINFEC), Instituto de Salud Carlos III, 28029 Madrid, Spain; 4 Consejo Superior de Investigaciones Científicas (CSIC), 28006 Madrid, Spain; 5 Facultad de Medicina, Universidad de Sevilla, 41004 Seville, Spain; 6 Unidad de Medicina Tropical, Hospital de Poniente, 04700 Almería, Spain; 7 Unidad de Gestión Clínica de Enfermedades Infecciosas y Microbiología, Hospital Universitario Virgen Macarena, 41009 Seville, Spain

**Keywords:** piperacillin/tazobactam, OPAT, drug delivery

## Abstract

**Objectives**: Piperacillin/tazobactam (P/T) is a broad-spectrum β-lactam antibiotic frequently used in outpatient parenteral antimicrobial therapy programs (OPAT). We aim to compare the clinical outcomes of P/T treatment in two models of OPAT care in order to maximize the utilization of health resources. **Material and methods**: We conducted a prospective observational study with retrospective analysis of a cohort of patients treated with P/T delivered every 24 or 48 h in an OPAT program. The primary outcomes were treatment failure during the OPAT episode and 30 day treatment failure. A bivariate and multivariate logistic regression model was built. A two-sided *p* < 0.05 was considered statistically significant. **Results**: Between 2012 and 2022, 247 patients were treated with P/T. Treatment was delivered daily in 176 patients (Group 24) and every two days in 71 patients (Group 48). The rate of treatment failure during OPAT in Group 24 and Group 48 was 12.4% (*n* = 22) and 5.6% (*n* = 4), respectively (*p* = 0.112); and the rate of treatment failure 30 days after OPAT treatment end was 18.2% (*n* = 32) and 21.1% (*n* = 15) in Group 24 and Group 48, respectively (*p* = 0.594). Treatment every 24 or 48 h was not associated with higher treatment failure during OPAT or 30 days after finishing OPAT in either bivariate or multivariate analysis. **Conclusions**: P/T administration in OPAT programs being replaced every two days is feasible without an increase in treatment failure, relapse, or mortality compared to daily drug replacement. These findings should motivate further research to facilitate the implementation of this novel delivery strategy in OPAT programs.

## 1. Introduction

Outpatient parenteral antimicrobial therapy (OPAT) programs aim to reduce hospital stays in patients requiring prolonged intravenous treatments without oral options, but with sufficient clinical stability to avoid future hospitalizations [1]. OPAT programs have proven to be cost-effective, linked to enhanced patients’ comfort, and reduced likelihood of healthcare-related complications [2,3].

In this context, various types of infusions (intermittent, extended, or bolus) are commonly administered by gravity or pumps, which also allows continuous infusion [4]. Infusion method is determined by factors such as the antimicrobial agent, patient’s condition, treatment goals, and program resources. Different OPAT models have been outlined, encompassing programs based in homes, clinics, and self-administration. The specific resources needed for each model vary, influencing the number and type of patients involved [5,6]. In Spain, OPAT models commonly involve daily home visits by nurses, and follow-up from a physician experienced in OPAT. This visit includes monitoring of clinical stability and administration of the drug [7]. This model requires daily involvement of the hospital pharmacy for drug preparation, which is delivered already diluted. The primary benefits of this model include ongoing monitoring of the patient by a healthcare professional, prompt identification of any adverse drug effects or progression of infections, and the assurance of consistent care provided by the same healthcare team.

Piperacillin/tazobactam (P/T) is a broad-spectrum β-lactam antibiotic commonly used in OPAT, offering a versatile treatment option for a wide range of infections [8]. The chemical stability and pharmacokinetic/pharmacodynamic properties of P/T allow its administration via continuous (24 h) or extended (3 h) infusion through an electronic or elastomeric pump stored at room temperature. This provides the potential for daily drug-replacement in a single visit, streamlining the treatment process. Moreover, its chemical stability allows the preparation of two daily doses in a single solution and its administration through a pump during 48 h, enabling the option to prepare and replace it every two days. This expanded timeframe allows for the inclusion of more patients in the OPAT program, while also enhancing cost-efficiency and resource allocation [9,10]. Achieving a harmonious equilibrium between operational efficiency and patients’ safety is crucial. Therefore, the aim of our study was to compare the clinical outcomes of P/T treatment replaced daily vs. each 48 h in an OPAT program in order to maximize the utilization of our health resources.

## 2. Materials and Methods

### 2.1. Study Design and Setting

We conducted a post hoc analysis of a prospective collected cohort of patients treated in an OPAT program shared by two tertiary hospitals. The key features of our OPAT program have been outlined in previous publications [9,10]. A multidisciplinary team determined patient inclusion for the program and antimicrobial treatment. All antibiotics were prepared from buffer-free authorized intravenous drug presentations by the pharmacy service under sterile conditions in a laminar flow cabinet. The STROBE guidelines for reporting observational studies were followed [11]. This program has the approval of the Ethics Committee for Clinical Research of the two hospitals involved.

### 2.2. Patient Population

Inclusion criteria comprised adult patients treated with P/T, prepared and replaced every 24 or 48 h in our OPAT program between August 2012 and December 2022 and patients included in OPAT with palliative intent (palliative OPAT). Patients on haemodialysis were excluded from the analysis. For those treated in the OPAT program more than once, only the first episode was included if the following episodes occurred within 30 days of the first inclusion, as they were considered recurrences.

Two models of OPAT care were included in our program for P/T treatments:

1. Daily assistance (group 24 h): Nurse visitation for clinical care and treatment delivery was settled daily. The complete daily dose of P/T was in a 500–750 mL 0.9% sodium chloride single solution under sterile condition at the pharmacy service. The concentration of P/T solution was in the range between 48/6 mg/mL and 64/8 mg/mL. Every day, the nurse replaced the P/T solution and programmed an electronic programmable infusion pump to administer an extended (3 h) infusion (2–4 g/6–8 h during 24 h). This model was available since the beginning of the program (2012).
2. Every-other-day assistance (48 h group): Nurse visitation for clinical care and treatment delivery was settled every two days. The complete two-daily dose of P/T was prepared in a 500–750 mL 0.9% sodium chloride single solution under sterile conditions at the pharmacy service. The concentration of P/T solution was in the range between 48/6 mg/mL and 64/8 mg/mL. Every two days, the nurse replaced the P/T solution and programmed an electronic programmable infusion pump to administer an extended (3 h) infusion (2–4 g/6–8 h during 48 h). This model has been available since 2018.

The assignment of treatment groups (daily delivery or every 48 h) depended on the clinician’s criteria (if the patient needed daily supervision or not), distance from home to hospital, and the available resources.

P/T stability data was thoroughly reviewed, and drug stability acceptance was confirmed by the OPAT team [12]. P/T stability data in 0.9% sodium chloride at room temperature have been previously studied between 20 and 125 mg/mL over a period of at least two days, using containers made of PVC, polypropylene, polyolefin, polyethylene, and elastomeric materials [13,14,15,16,17,18,19]. The use of electronic pumps (not in contact with the patient’s body) allows the use of room temperature stability data. Regardless, we instructed our patients to maintain proper room conditions to favor drug stability (and ensured it is correct in each nurse visitation).

### 2.3. Data Collection and Definitions

Data collected include sex, age, Charlson comorbidity index (CCI), comorbidities, type of infection, type of vascular access, infection acquisition, duration of treatment (during hospital admission and during OPAT), clinical outcomes (unplanned readmission, infection relapse or progression, or death during OPAT and 30 days after treatment end), and adverse events. Period of OPAT inclusion (2012–2017 vs. 2018–2022) was recorded due to internal changes in the venous access (prioritizing midline catheter) and the option of delivery every 48 h. Infection acquisition was categorized based on the criteria from the Center for Disease Control and Prevention [20]. The primary outcomes were treatment failure during the OPAT episode (a composite endpoint defined as unplanned readmission, infection progression, or death) and 30 day treatment failure [a composite endpoint retrospectively collected and defined as unplanned readmission (related or not to prior infection) or death during 30 days of follow-up after completion of OPAT treatment]. The secondary outcomes were adverse events and catheter related complications.

### 2.4. Statistical Analysis

Initially, a descriptive analysis was performed. Categorical variables were presented as percentages, while continuous variables were reported as the median and interquartile range (IQR). The Mann–Whitney *U* test was used to compare quantitative variables, and categorical variables were compared using the Chi-square test or Fisher’s exact test. Subsequently, factors associated with treatment failure during the OPAT episode and at 30 days were evaluated through both bivariate and multivariate analyses. In the multivariate analysis, a logistic regression model was applied to identify factors associated with treatment failure, calculating odds ratios (ORs), and 95% confidence intervals (CIs). We included all the explicative variables that are clinically relevant or previously associated with poor outcomes. A two-sided *p* < 0.05 was considered statistically significant. No data were missing from the multivariate model.

The statistical analysis was performed using SPSS version 28.0 (SPSS Inc., Chicago, IL, USA).

## 3. Results

### 3.1. Population and Treatment Characteristics

Between 2012 and 2022, 1834 patients were included in the OPAT program, preventing 24,110 days in hospital. Among them, 247 (13.5%) were treated with P/T, avoiding 3502 days of hospitalization. Treatment was delivered daily to 176 patients (Group 24) and every two days to 71 patients (Group 48). Baseline characteristics of patients included in both groups (Table 1) were similar, despite the prevalence of malignancies, being more common in Group 48 (27.8% vs. 42.3%, *p* = 0.028).

Infections treated with P/T in this cohort were heterogeneous. The most frequent type of infection was intra-abdominal or anorectal infection with or without abscess (34.8%). No statistically significant difference related to the type of infection was found among the two treatment groups. The most common Gram-negative microorganisms isolated in monomicrobial infections were *Pseudomonas aeruginosa* (44.2%) and *Escherichia coli* (18.3%). No differences were found among the treatment groups regarding the causal agent.

Treatment characteristics were similar between both groups, as shown in Table 2. Six patients continued with oral sequential antibiotic treatment after finishing OPAT, five of whom were in Group 48. The most common vascular access was peripheral access in Group 24 and midline catheter in Group 48 (73.9% and 71.8%, respectively). Contact isolation was required for 24 patients (9.7%).

### 3.2. Clinical Outcomes

The main clinical outcomes are summarized in Table 2. The rate of treatment failure during OPAT in Group 24 and Group 48 was 12.4% (*n* = 22) and 5.6% (*n* = 4), respectively (*p* = 0.112); and the rate of treatment failure 30 days after OPAT treatment end was 18.2% (*n* = 32) and 21.1% (*n* = 15) in Group 24 and Group 48, respectively (*p* = 0.594).

The causes of treatment failure during OPAT among the patients in Group 24 were unplanned readmission (68.2%, *n* = 15), infection progression that did not require readmission (22.7%, *n* = 5), and death (9%, *n* = 2). Causes of readmission were infection progression (60%, *n* = 9), non-infection-related disease cause (20%, *n* = 3), side effects from antibiotics (hepatitis and fever) (13.3%, *n* = 2), and scheduled surgical procedure (6.7%, *n* = 1). Two patients had allergic reactions that did not require hospitalization. Two patients died during OPAT at their homes because of infection progression that required palliative support. Regarding Group 48, all episodes of treatment failure during OPAT were attributed to unplanned readmissions (5.6%, *n* = 4). In this group, one of the patients was readmitted to control the infectious focus, and in the other three cases, the readmission was not related to the infectious process. There were no infection progressions (besides the patient readmitted to control the infectious focus) or deaths in Group 48.

Regarding causes of 30 day failure after the end of OPAT treatment, cases of readmission related to prior infection were 9.1% (*n* = 16) and 14.1% (*n* = 10) in Group 24 and Group 48, respectively. Nine patients died within 30 days, eight in Group 24 (4.5%) and one in Group 48 (1.4%) (*p* = 0.453). No statistically significant differences were found regarding 30-day failure after the end of OPAT treatment, unplanned readmission or death between the groups.

The rate of catheter-related complications in this cohort was 27.9%. There was a significant increase in these complications in Group 24 with respect to Group 48 (33% vs. 15.5%, *p* = 0.006). The main cause of these complications was extravasation, malfunction, or catheter loss in both groups (19.9% vs. 14.1% in Group 24 and Group 48 *p* = 0.285, respectively). Phlebitis occurred in 24 patients, 23 in Group 24, and 1 in Group 48 (13.1% vs. 1.4%, *p* = 0.004).

### 3.3. Bivariate and Multivariate Analysis Model

The results of the bivariate are shown in Supplementary Tables S1 and S2 and multivariate analyses are shown in Table 3 and Table 4. The treatment group was not associated with treatment failure during OPAT or 30 days after finishing OPAT in either bivariate or multivariate analysis.

## 4. Discussion

P/T is a cornerstone in the treatment of several bacterial infections [8]. In this study, we presented the implementation of a novel P/T OPAT model, which harnesses resource allocation and maximizes the number of patients who could benefit from home treatment. Drug delivery and replacement every two days achieved similar clinical outcomes to daily delivery while utilizing half the resources.

Historically, antibiotics administered once daily in a short infusion have been mainly used in OPAT programs because they could be easily adapted to different OPAT models and do not require complex equipment or support [2,4,5,21,22]. This has contributed to an overuse of cephalosporins and carbapenems [7]. On the other hand, multiple daily dose administration requires a more complex structure. One option is to instruct patients or caregivers on self-administration of antimicrobials at home. This is only appropriate for skilled patients in a suitable environment, as it involves multiple catheter and intravenous drug manipulations in a non-sterile setting [5,6]. Another option is drug administration by a healthcare professional, which ensures continuous expert supervision and early detection of adverse effects or infection progression. Drug administration at home or in a healthcare facility could be arranged by multiple daily visitations, which increases resource and time consumption, or by one daily visitation, which is more comfortable for patients and more efficient for the healthcare system [4,5,6]. In our case, the total daily dose is prepared in the hospital pharmacy in a sterile area as a single solution and drug administration is arranged during the day using programmable electronic pump or elastomeric pumps. The use of programmable pumps is a versatile option which allows for the administration of single or multiple daily doses as intermittent, prolonged, or continuous infusions with minimal nurse time consumption (only a short visitation for drug solution replacement and pump programming). Other alternatives such as premixed single-dose solutions (commercial or not) have been proposed. However, they require patient or caregiver catheter manipulation (as previously discussed) and specific storage conditions, which hinder universal access. Our model widens patient selection; however, it requires concrete stability data [18,23].

In our study, we investigated beyond this model presenting the longest cohort of patients treated with P/T with daily replacement of the medication and healthcare supervision, and compared the clinical results with a novel optimized delivery program in which the two-day dose was prepared in a single solution and the drug was replaced every two days. The implementation of this administration model was feasible due to the previously mentioned stability studies and the enhanced expertise of our staff in mid-line catheter placement. Similar clinical outcomes were achieved in both cohorts and none of the delivery methods were associated with worse prognosis, which is promising for the planning of OPAT programs. To the best of our knowledge, only one study has previously presented this strategy [10].

The administration of P/T via OPAT has been previously reported across various OPAT programs [7,21,22,23,24,25,26,27,28]. Five of them [24,25,26,27,28] administered P/T in continuous infusion using elastomeric devices containing one daily dose; two studies reported outcomes from various administration models [21,22], and only one [23] used electronic programmable pumps for the administration of a single solution containing one daily dose. One of the studies did not specify the administration method [7]. None of the previous reports of OPAT P/T administration extended the timeframe of drug replacement beyond daily delivery. In our experience, electronic programmable pumps seem to be a more flexible option rather than elastomeric pumps, as they allow for intermittent or continuous infusion and are not limited by a fixed volume, unlike elastomeric devices. In addition, high volume solutions can be administered through electronic pumps, allowing the dilution of the two-day dose without compromising chemical stability. Concerning clinical outcomes of patients treated in OPAT programs with P/T, most of the studies reported global outcomes of the OPAT program without itemizing by drug; therefore, results are not comparable with ours [7,21,22,23,24,25,27,28]. One study [26] reported 35 *P. aeruginosa* infections treated with P/T in OPAT. Among them, infection symptoms were resolved in 93% of patients at the end of the treatment, which is similar to the clinical success rate during OPAT observed in both Group 24 and Group 48 (87.5% and 94.4%, respectively). The assessment of treatment failure 30 days after treatment completion is not common in similar OPAT studies published [7,21,24,28]. In those studies that analyzed it, the failure rare oscillates between 5% [25,26] and 25% [22,23,27]. In those studies, the definition of treatment failure was heterogeneous, with some considering only readmission or death related to the prior infection, while others included all-cause outcomes. In our case, the definition of the variable 30-day treatment failure is quite strict and included all causes of unplanned readmission or death, yielding results similar to those of other reports (19% 30-day treatment failure). Vascular access complication was markedly higher in our study (8% vs. 27.9%), mainly compared to the Group 24 rate (33%). It might be explained due to the initial predominant use of peripheral catheters in our OPAT program.

Our study has several limitations. First, the observational prospective design, but retrospective analysis implies an inherent risk of bias. Second, a change in the vascular catheter access policy took place during the study period, which worsened Group 24’s safety results. Third, group selection was at the discretion of the responsible clinical, which introduced a bias of selection. Fourth, therapeutic drug monitoring of P/T was not implemented in our program, which might have hindered the detection of fluctuations in drug exposition in each OPAT model. Fifth, a propensity score matching was not considered necessary due to the similar characteristics of the two arms. Lastly, follow-up in charge of the OPAT team was similar for all infections, despite a longer follow-up being provided by the clinician in charge in some cases.

The main strength of this study is its real-life framework that supports the viability of this OPAT model. It might be applicable to other antibiotics. The close follow-up evaluations and nurse visitations of all patients included in both OPAT models promote early detection of complications.

In conclusion, the results of our study show that P/T administration in OPAT programs being replaced every two days is feasible without an increase in treatment failure, relapse, or mortality compared to daily drug replacement. These findings should motivate further research to broaden the evidence and facilitate the implementation of this novel delivery strategy in more OPAT programs.

## Figures and Tables

**Table 1:** Baseline and infection characteristics.

			All Cases (*n* = 247)	Group 24 (*n* = 176)	Group 48 (*n* = 71)	*p* Value ^a^
**Baseline Characteristics**						
Median age (IQR)			63 (49–72)	62 (48–72)	63 (51–71)	0.644 ^b^
Male gender			159 (64.4%)	115 (65.3%)	44 (62.0%)	0.617
Comorbidities						
	Median Charlson score (IQR)		2 (1–3)	2 (1–3)	2 (1–4)	0.638
	Diabetes mellitus		48 (19.4%)	35 (19.9%)	13 (18.3%)	0.777
	COPD		61 (24.7%)	48 (27.3%)	13 (18.3%)	0.139
	Chronic renal failure		26 (10.5%)	17 (9.7%)	6 (8.5%)	0.767
	Chronic heart failure		60 (24.3%)	39 (22.2%)	21 (29.6%)	0.219
	Malignancy		79 (32.0%)	49 (27.8%)	30 (42.3%)	0.028
	Chronic liver disease		23 (9.3%)	15 (8.5%)	8 (11.3%)	0.502
**Related to Infection**						
Acquisition of infection						
	Community-acquired		168 (68.0%)	125 (71.0%)	43 (60.6%)	0.111
	Nosocomial/healthcare acquisition		79 (31.9%)	51 (29.0%)	28 (39.4%)	0.111
Department						
	Surgery		57 (23.1%)	41 (23.3%)	16 (22.5%)	0.898
	Medical		190 (76.9%)	135 (76.7%)	55 (77.5%)	0.898
		Infectious diseases inpatient setting	51 (20.6%)	35 (19.9%)	16 (22.5%)	0.642
		Infectious diseases outpatient setting	24 (9.7%)	13 (7.4%)	11 (15.4%)	0.052
		Pneumology inpatient setting	42 (17.0%)	35 (19.9%)	7 (9.9%)	0.058
		Pneumology outpatient setting	6 (2.4%)	4 (2.3%)	2 (2.8%)	1.000 ^c^
		Internal Medicine	24 (9.7%)	17 (9.7%)	7 (9.9%)	0.962
		Oncology and Hematology	20 (8.1%)	11 (6.3%)	9 (12.7%)	0.094
		Others	23 (9.3%)	20 (11.4%)	3 (4.2%)	0.081
Diagnosis						
	Intra-abdominal or anorectal infection or abscess		86 (34.8%)	59 (33.5%)	27 (38%)	0.501
	Pneumonia		22 (8.9%)	14 (8.0%)	8 (11.3%)	0.408
	Exacerbated COPD		11 (4.5%)	9 (5.1%)	2 (2.8%)	0.734 ^c^
	Bronchiectasis		33 (13.4%)	28 (15.9%)	5 (7.0%)	0.064
	Lung abscess		22 (8.9%)	16 (9.1%)	6 (8.5%)	0.873
	Complicated Urinary Tract Infection		27 (10.9%)	18 (10.2%)	9 (12.7%)	0.577
	Skin and soft tissue infection		16 (6.5%)	11 (6.3%)	5 (7.0%)	0.781 ^c^
	Endovascular infection		11 (4.5%)	9 (5.1%)	2 (2.8%)	0.734 ^c^
	Osteoarticular infection		15 (6.1%)	10 (5.7%)	5 (7.0%)	0.769
	Others		4 (1.6%)	2 (1.1%)	2 (2.8%)	0.326 ^c^
Microbiological isolation						
	None		94 (38.1%)	65 (36.9%)	29 (40.8%)	0.566
	Polymicrobial		48 (19.4%)	34 (19.3%)	15 (21.1%)	0.747
	Monomicrobial					
		*Pseudomonas aeruginosa*	46 (18.6%)	33 (18.8%)	13 (18.3%)	0.936
		*Escherichia coli*	19 (7.7%)	15 (8.5%)	4 (5.6%)	0.441
		*Klebsiella* spp.	5 (2.0%)	3 (1.7%)	2 (2.8%)	0.627
		Other Gram-negative bacilli	19 (7.7%)	15 (8.5%)	4 (5.6%)	0.441
		Gram-positive cocci (*Streptococcus* spp. and *Enterococcus* spp.)	15 (6.1%)	11 (6.3%)	4 (5.6%)	1.000 ^b^

Data are presented as *n* (%) unless indicated otherwise. Abbreviation: IQR—interquartile range; COPD—chronic obstructive pulmonary disease. ^a^ *p* values were calculated by Chi-square test, except where otherwise specified. ^b^ Mann–Whitney *U*-test. ^c^ Fisher test.

**Table 2:** Treatment characteristics and clinical outcomes.

			All Cases (*n* = 247)	Group 24 (*n* = 176)	Group 48 (*n* = 71)	*p* Value ^a^
**Treatment Characteristics**						
Length of hospital stay in days, median (IQR)			10 (6–17)	9 (6–16)	11 (7–21)	0.456
Median inpatient treatment days (IQR)			7 (4–10)	6.5 (4–9)	7 (4–10)	0.545
Median OPAT treatment days (IQR)			11 (7–17)	11 (7–16)	10 (6–21)	0.309
Period of inclusion (2018–2022)			99 (40.1%)	28 (15.9%)	71 (100%)	<0.001
Vascular access						
	Peripheral access		140 (56.7%)	130 (73.9%)	10 (14.1%)	<0.001
	Central access with peripheral insertion		21 (8.5%)	14 (8.0%)	7 (9.9%)	0.627
	Midline catheter		75 (30.4%)	24 (13.6%)	51 (71.8%)	<0.001
	Central access		4 (1.6%)	4 (2.3%)	0	0.581
	Reservoir		7 (2.8%)	4 (2.3%)	3 (4.2%)	0.413
**Clinical Outcomes**						
During OPAT time						
	Treatment failure		26 (10.5%)	22 (12.4%)	4 (5.6%)	0.112
		Unplanned readmission	19 (7.7%)	15 (8.5%)	4 (5.6%)	0.441
		Infection progression	5 (2.0%)	5 (2.8%)	0	0.325 ^b^
		Death during OPAT	2 (0.8%)	2 (1.1%)	0	1.000 ^b^
	Vascular access complication		69 (27.9%)	58 (33.0%)	11 (15.5%)	0.006
		Phlebitis	24 (9.7%)	23 (13.1%)	1 (1.4%)	0.004 ^b^
		Extravasation, malfunction or catheter loss	45 (18.2%)	35 (19.9%)	10 (14.1%)	0.285
30 day treatment failure (composite endpoint)			47 (19.0%)	32 (18.2%)	15 (21.1%)	0.594
	30 day readmission (related to prior infection)		26 (10.5%)	16 (9.1%)	10 (14.1%)	0.247
	30 day readmission (non-related to prior infection)		12 (4.9%)	8 (4.5%)	4 (5.6%)	0.747 ^b^
	Death		9 (3.6%)	8 (4.5%)	1 (1.4%)	0.453 ^b^

Data are presented as *n* (%) unless indicated otherwise. Abbreviation: IQR—interquartile range; OPAT—Outpatient parenteral antimicrobial treatment. ^a^ *p* values were calculated by Chi-square test, except where otherwise specified. ^b^ Fisher test.

**Table 3:** Multivariate logistic regression analysis of variables associated with treatment failure during OPAT treatment.

	*p* Value ^a^	Adjusted OR
Malignancy	0.809	1.12 (0.45–2.76)
Outpatient setting	0.294	0.29 (0.37–2.33)
Intra-abdominal or anorectal infection or abscess	0.304	0.61 (0.24–1.56)
Midline catheter	0.153	0.35 (0.08–1.48)
Treatment group 48 h	0.691	0.77 (0.21–2.86)

^a^ *p* values were calculated by Chi-square test, except where otherwise specified.

**Table 4:** Multivariate logistic regression analysis of variables associated with 30-day treatment failure.

	*p* Value ^a^	Adjusted OR
Malignancy	<0.001	3.30 (1.63–6.67)
Chronic liver disease	0.006	3.84 (1.46–10.06)
*Pseudomonas aeruginosa*	0.017	2.56 (1.18–5.54)
Midline catheter	0.609	0.79 (0.32–1.93)
Treatment group 48 h	0.765	0.87 (0.36–2.14)

^a^ *p* values were calculated by Chi-square test, except where otherwise specified.

## Data Availability

The data presented in this study are available on request from the corresponding author due to privacy and ethical reasons.

## Supplementary Materials

The following supporting information can be downloaded at: https://www.mdpi.com/article/10.3390/pharmaceutics17111429/s1, Table S1: Bivariate analysis of variables associated with treatment failure during OPAT treatment; Table S2: Bivariate analysis of variables associated with 30-day treatment failure.
