# Supplementary material for: Clinical Outcomes of Piperacillin/Tazobactam Treatment in Outpatient Parenteral Antimicrobial Therapy (OPAT) Programs: Comparison of Two Models of Opat Care
Source: Pharmaceutics. 2025 Nov 4;17(11):1429. doi: 10.3390/pharmaceutics17111429 (PMC12655769; doi:10.3390/pharmaceutics17111429)
Supplement: Supplementary file 1 [file pharmaceutics-17-01429-s001.zip › pharmaceutics-3872084-supplementary.pdf]

pharmaceutics

MDPI

Supplementary Materials

Table S1. Bivariate analysis of variables associated with treatment failure during OPAT treatment

Table S2. Bivariate analysis of variables associated with 30-day treatment failure

Supplementary Materials

Table S1. Bivariate analysis of variables associated with treatment failure during OPAT treatment.

	Treatment success during OPAT time (n = 221)	Treatment failure during OPAT time (n = 26)	p value[a]	OR (95% CI)
**Baseline Characteristics**				
Median age (IQR)	63 (49 - 72)	63 (44 - 69)	0.669[b]	1.00 (0.97 – 1.02)
Male gender	140 (63.3%)	19 (73.1%)	0.327	1.57 (0.63 – 3.90)
Comorbidities				
Median Charlson score (IQR)	2 (1 - 3)	2 (1 - 3)	0.927[b]	0.99 (0.81 – 1.21)
Diabetes mellitus	43 (19.5%)	5 (19.2%)	0.978	0.99 (0.35 – 2.76)
COPD	51 (23.1%)	10 (38.5%)	0.085	2.08 (0.89 – 4.87)
Chronic renal failure	22 (10.0%)	1 (3.8%)	0.484[c]	0.36 (0.05 – 2.80)
Chronic heart failure	54 (24.4%)	6 (23.1%)	0.879	0.93 (0.35 – 2.43)
Malignancy	70 (31.7%)	9 (34.6%)	0.761	1.14 (0.49 – 2.69)
Chronic liver disease	20 (9.0%)	3 (11.5%)	0.719[c]	1.31 (0.36 – 4.75)
**Related to infection**				
Nosocomial/healthcare acquisition	74 (33.5%)	5 (19.2%)	0.140	0.47 (0.17 – 1.30)
Department				
Surgery	49 22.2%)	8 (30.8%)	0.325	1.56 (0.64 – 3.80)
Medical				
Infectious diseases inpatient setting	48 (21.7%)	3 (11.5%)	0.308[c]	0.47 (0.14 – 1.63)
Infectious diseases outpatient setting	23 (10.4%)	1 (3.8%)	0.485[c]	0.34 (0.05 – 2.66)
Pneumology inpatient setting	36 (16.3%)	6 (23.1%)	0.408[c]	1.54 (0.58 – 4.11)
Pneumology outpatient setting	6 (2.7%)	0	1.000[c]	-
Internal Medicine	21 (9.5%)	3 (11.5%)	0.726[c]	1.24 (0.34 – 4.49)
Oncology and Hematology	18 (8.1%)	2 (7.7%)	1.000[c]	0.94 (0.21 – 4.30)
Others	20 (9.0%)	3 (11.5%)	0.719[c]	1.31 (0.36 – 4.75)
Diagnosis				
Intra-abdominal or anorectal infection or abscess	79 (35.7%)	7 (26.9%)	0.514[c]	0.66 (0.27-1.64)
Pneumonia	21 (9.5%)	1 (3.8%)	0.485[c]	0.38 (0.05 – 2.96)
Exacerbated COPD	10 (4.5%)	1 (3.8%)	1.000[c]	0.84 (0.10 – 6.87)
Bronchiectasis	29 (13.1%)	4 (15.4%)	0.761[c]	1.20 (0.39 – 3.74)
Lung abscess	18 (8.1%)	4 (15.4%)	0.265[c]	2.05 (0.64 – 6.60)
Complicated Urinary Tract Infection	23 (10.4%)	4 (15.4%)	0.502	1.57 (0.50 – 4.94)
Skin and soft tissue infection	15 (6.8%)	1 (3.8%)	1.000[c]	0.55 (0.07 – 4.34)
Endovascular infection	10 (4.5%)	1 (3.8%)	1.000[c]	0.84 (0.10 – 6.87)
Osteoarticular infection	13 (5.9%)	2 (7.7%)	0.663[c]	1.33 (0.28 – 6.27)
Others	3 (1.4%)	1 (3.8%)	0.361	2.91 (0.29 – 29.01)
Microbiological isolation				
None	88 (39.8%)	6 (23.1%)	0.096	0.45 (0.18 – 1.17)
Polymicrobial	44 (19.9%)	5 (19.2%)	1.000[c]	0.96 (0.34 – 2.68)
Monomicrobial				

Pseudomonas aeruginosa	39 (17.6%)	7 (26.9%)	0.286[c]	1.72 (0.68 – 4.37)
Escherichia coli	18 (18.1%)	1 (3.8%)	0.702[c]	0.45 (0.06 – 3.53)
Klebsiella spp.	5 (2.3%)	0	1.000[c]	-
Other Gram Negative bacilli	16 (7.2%)	3 (11.5%)	0.433[c]	1.67 (0.45 – 6.17)
Gram Positive cocci (*Streptococcus* spp. and *Enterococcus* spp.)	11 (5.0%)	4 (5.4%)	0.059[c]	3.471 (1.02 – 11.83)
**Treatment Characteristics**				
Period of inclusion (2018-2022)	92 (41.6%)	7 (26.9%)	0.148	0.52 (0.21-1.28)
Vascular access				
Peripheral Access	123 (55.7%)	17 (65.4%)	0.344	1.51 (0.64 – 3.52)
Central access with peripheral insertion	18 (8.1%)	3 (11.5%)	0.557	1.47 (0.40 – 5.38)
Midline catheter	72 (32.6%)	3 (11.5%)	0.027	**0.27 (0.08 – 0.93)**
Central access	1 (0.5%)	3 (11.5%)	0.004[c]	**28.70 (2.87 – 200.24)**
Reservoir	7 (3.2%)	0	1.000[c]	-
Treatment group 48h	67 (30.3%)	4 (15.4%)	0.112	0.42 (0.14-1.26)

Data are presented as n (%) unless indicated otherwise. Abbreviation: IQR – interquartile range; OPAT – Outpatient parenteral antimicrobial treatment. [a] *P* values were calculated by chi-square test, except where otherwise specified. [b] Mann-Whitney *U*-test. [c] Fisher test.

Table S2. Bivariate analysis of variables associated with 30-day treatment failure.

	30-day Treatment success (n = 200)	30-day Treatment failure (n = 47)	p value[a]	OR
**Baseline Characteristics**				
Median age (IQR)	63 (48 - 72)	63 (53 - 72)	0.972[b]	1.00 (0.98 – 1.02)
Male gender	76 (38.0%)	12 (25.5%)	0.108	1.79 (0.87 – 3.66)
Comorbidities				
Median Charlson score (IQR)	2 (1 - 3)	3 (1 - 5)	0.003	1.38 (1.11 – 1.47)
Diabetes mellitus	38 (19.9%)	10 (21.3%)	0.723	1.15 (0.53 – 2.52)
COPD	45 (22.5%)	16 (34.0%)	0.099	1.78 (0.89 – 3.54)
Chronic renal failure	22 (11.0%)	1 (2.1%)	0.090[c]	0.18 (0.02 – 1.34)
Chronic heart failure	50 (25.0%)	10 (21.3%)	0.592	0.81 (9.38 – 1.75)
Malignancy	55 (27.5%)	24 (51.1%)	0.002	2.75 (1.44 – 5.27)
Chronic liver disease	14 (7.0%)	9 (19.1%)	0.021	3.15 (1.27 – 7.80)
**Related to infection**				
Nosocomial/healthcare acquisition	64 (32.0%)	15 (31.9%)	0.991	1.00 (0.50 – 1.97)
Department				
Surgery	51 (25.5%)	6 (12.8%)	0.062	0.43 (0.17 – 1.07)
Medical				
Infectious diseases inpatient setting	42 (21.0%)	9 (19.1%)	0.778	0.89 (0.40 – 1.99)
Infectious diseases outpatient setting	21 (10.5%)	3 (6.4%)	9.584[c]	0.58 (0.17 – 2.04)
Pneumology inpatient setting	35 (17.5%)	7 (14.9%)	0.669	0.83 (0.34 – 1.99)

Pneumology outpatient setting	6 (3.0%)	0	0.598[c]	-
Internal Medicine	13 (6.5%)	11 (23.4%)	0.001[c]	4.40 (1.83 – 10.58)
Oncology and Hematology	15 (7.5%)	5 (10.6%)	0.551	1.47 (0.51 – 4.26)
Others	17 (8.5%)	6 (12.8%)	0.402	1.58 (0.59 – 4.24)
Diagnosis				
Intra-abdominal or anorectal infection or abscess	70 (35.0%)	16 (34.0%)	1.000[c]	0.96 (0.49-1.87)
Pneumonia	15 (7.5%)	7 (14.9%)	0.150[c]	2.16 (0.83 – 5.64)
Exacerbated COPD	8 (4.0%)	3 (6.4%)	0.443[c]	1.64 (0.42 – 6.42)
Bronchiectasis	26 (13.0%)	7 (14.9%)	0.731	1.17 (0.75 – 2.89)
Lung abscess	18 (9.0%)	4 (8.5%)	1.000[c]	0.94 (0.30 – 2.92)
Complicated Urinary Tract Infection	23 (11.5%)	4 (8.5%)	0.555	0.72 (0.24 – 2.18)
Skin and soft tissue infection	16 (8.0%)	0	0.047[c]	-
Endovascular infection	7 (3.5%)	4 (8.5%)	0.228[c]	2.57 (0.72 – 9.15)
Osteoarticular infection	13 (6.5%)	2 (4.3%)	0.743[c]	0.64 (0.14 – 2.93)
Others	4 (2.0%)	0	1.000[c]	-
Microbiological isolation				
None	86 (43.0%)	8 (17.0%)	0.001	0.27 (0.12 – 0.61)
Polymicrobial	35 (17.5%)	14 (29.8%)	0.057	2.00 (0.97 – 4.12)
Monomicrobial				
Pseudomonas aeruginosa	32 (16.0%)	14 (29.8%)	0.029	2.23 (1.07 – 4.62)
Escherichia coli	16 (8.0%)	3 (6.4%)	1.000[c]	0.78 (0.22 – 2.81)
Klebsiella spp.	4 (2.0%)	1 (2.1%)	1.000[c]	1.07 (0.12 – 9.76)
Other Gram Negative Bacillus	15 (7.5%)	4 (8.5%)	0.765[c]	1.15 (0.36 – 3.63)
Gram Positive cocci (*Streptococcus* spp. and *Enterococcus* spp.)	12 (6.0%)	3 (6.4%)	1.000[c]	1.09 (0.29 – 3.95)
**Treatment Characteristics**				
Period of inclusion (2018-2022)	77 (38.5%)	22 (46.8%)	0.296	1.40 (0.74-2.66)
Vascular access				
Peripheral Access	118 (59.0%)	22 (46.8%)	0.129	0.61 (0.32 – 1.16)
Central access with peripheral insertion	15 (7.5%)	6 (12.8%)	0.250[c]	1.81 (0.66 – 4.93)
Midline catheter	60 (30.0%)	15 (31.9%)	0.797	1.09 (0.55 – 2.17)
Central Access	2 (1.0%)	2 (4.3%)	0.165[c]	4.40 (0.60 – 32.08)
Reservoir	5 (2.5%)	2 (4.3%)	0.621[c]	1.73 (0.33 – 9.22)
Treatment group 48h	56 (28.0%)	15 (31.9%)	0.594	1.21 (0.61 – 2.40)

Data are presented as n (%) unless indicated otherwise. Abbreviation: IQR – interquartile range; OPAT – Outpatient parenteral antimicrobial treatment. [a] *P* values were calculated by chi-square test, except where otherwise specified. [b] Mann-Whitney *U*-test. [c] Fisher test.
